# The Brain Power of Man

**Published:** 1874-07

**Authors:** 


### THE BRAIN POWER OF MAN.

### HAS HE TWO BRAINS? OR HAS HE ONLY ONE?

*Dr. Brown-Sequard's Theories—He claims that we have two Brains—One on the right side and the other on the left—the latter nurtured to the neglect of the former—Both should be educated to perfect mental and physical development.*

Have we two brains? and, if so, why not educate both? The views of science upon this subject were different from his. The left side of the body, was the side affording volition to the brain, and, *vice versa*, the right side of the brain afforded volition to the body. Eminent authorities had declared that either side of the brain was competent for this purpose.

But we use only one side, and, therefore, leave out of account one half of brain matter. We owe due education to both sides of the brain, or, rather, to the two brains.

As to intelligence, the eminent authorities he had cited established the fact that either side of the brain was competent for full development of the faculties. There were many persons of two minds, because they were never able to make up their minds. Some men claim to be rational while they are insane. There were many cases that show clearly that there were two brains He had known a boy in London that manifestly had two brains, whose peculiarities he described. He would fall into a comatose state, and suddenly open his eyes brightly, inquiring of his mother why he was not introduced to the gentleman who was present while he was asleep. Again, the lecturer saw him when the boy recognized him. He had two mental lives. He knew nothing of what occurred in his sleeping condition, when fully awake; and when in the latter condition he knew what had occurred when in the former. The lecturer had seen three cases of this kind.

As regards faculty of speech, the fact that we had two brains was not so easily proved. The loss of the faculty of expression depends upon disease of the left side of the brain; and this proves that the right side is distinct.

As regards sight a theory has been put forth by a celebrated physician of London that the right side of the base of the brain is the centre of sight. The inner half of the right eye and the outer half of the left eye have the base of the brain as the center. A disease in the left side of the brain, where the optic nerve touches, would therefore affect only one-half of the brain. Notable cases were given in which parties had seen but one-half of certain objects that they gazed upon. If the disease exists only in the left side of the base of the brain, only one-half of the eye will be affected. So there are many cases that go to sustain the philosophers. But we do not accept conclusions unless theory is thoroughly supported.

There were three series of facts, but one would be enough, to show that the theory should be rejected. Disease of the brain, where the optic nerve touches, would not be sufficient to cause loss of sight. One side of the brain would be sufficient to sustain sight. An alteration in any portion of the nervous system, acting upon other parts, can produce disease in that part. Injury to the spinal cord would produce loss of sight on either side. There was nothing more common than the loss of sight temporarily in children who suffered from worms in the stomach. Any injury in one-half of the brain can exist without producing loss of sight. Either half of the brain may, therefore, serve to sustain sight.

As to the voluntary movements, these depended upon the action of the body. Yet there were many small muscles which were not affected in cases of paralysis. There were cases on record in which it was shown that the lower lobe of the brain could be destroyed without affecting these voluntary movements. There were several such cases. We must, therefore, look on one-half of the brain as being sufficient to sustain voluntary movements on both sides of the body. An irritation in any part of the brain may affect any part of the body, and an irritation in any part of the body can produce paralysis in another part. The irritation could also act upon remote parts. This shows that the power of will does not control the entire actions of the body. When paralysis occurs it depends upon irritation.

The same reasoning applies to sensation. There were thousands of cases affecting the brain that did not affect the feeling. Passing these facts in review we find vast differences owing to the fact that one-half of the brain was developed for certain things and the other half for other things. To the left side of the brain belonged the faculty of expressing ourselves by speech. Articulation depended in great measure upon the left side of the brain. Difficulties in the mechanical point of speech were more frequently found when the left side of the brain was diseased. It was the mental part that was lost, and not the mere mechanical action. The left side of the brain was also the motive power of gesture. When the left side was diseased patients lost the power of gesticulation

As regards writing, it was lost more frequently in diseases of the left side of the brain. The right arm was paralyzed by diseases of this side. Many thus diseased could not write from memory, although they could use their fingers and copy. In those cases it sometimes occurs that persons could not write at all.

Intelligence depends more upon the healthfulness of the left side than of the right side of the brain. The right side of the brain in some cases has the power of the left, if properly developed. This serves to hysterical developments and to nutrition of the body. One, the left, applies to mental; the other, to the natural life.

The right side of the brain operates upon the limbs in cases of paralysis and other diseases; also upon disturbances in the lungs, liver and other parts. Hysterical and emotional symptoms are more common in cases of disease of the right side of the brain: out of 120 cases of paralysis that came under the lecturer's observation there were 96 caused by disease of the right side. An alteration of the retina of the eye will come more frequently from diseases of this side of the brain. Out of 69 cases of convulsions of the eyes 47 were due to disease of the right side. Death occurs much more frequently by diseases of the right side of the brain, and in cases where patients do not die it will produce more extensive and enduring paralysis.

All this, shows not that the two sides of the brain differed originally, but that there were different developments of each. The left side of the brain was much larger than the right side. If a person went frequently to the same hatter, he would find that his hat had from time to time to be enlarged. There was no question that the brain grew. By studying a particular subject the person became more proficient, and the brain was more fully developed.

There was no doubt that the left side of the brain predominated in our system. Our being right-handed showed it. There was no population in the world that was not right-handed. The right hand of the body was mostly used.

Left-handed individuals used the right side of the brain, showing the connection between these things.

There was primitively a difference between the two brains. In children convulsion were sooner developed in the left than in the right side of the brain. This was attributable to excess of blood in the left side. Parrots roosted on the right legs, and their talking power came from the left side of the head.

There were four vital points to be considered. The first was that asphyxia was connected with the left side of the brain in persons that were right-handed, and with the right side in those that were left-handed. The second point was that children who were first learning to talk, if disease came in the left side of the brain, learned to talk just as well with the right side of the brain. Though losing half of the brain they got along just as well.

This proved that the right side could be educated, with the left hand for execution. The third point was, that four out of every hundred left-handed persons learned to write with the left hand; therefore the left side of brain, even with persons left-handed, could be educated better than the right side. The fourth point was that the leg was rarely ever so much affected by paralysis as the arm. He however would pass over this argument, as it could only be understood by medical men.

If the lecturer had established that we had two brains then they should be developed. If we could develop the legs and arms of both sides we could develop both sides of the brain. If we gave as much attention to the left side of the body as we do to the right side we would fully develop our two brains. The important point, therefore, would be to make children use both sides of the body—alternately using the right and left arm and the right and left leg equally. There would be no difficulty in thus training children to full development.

Even adults who had lost speech by disease of the left side of the brain could regain the power by cultivating the right side. In gesture, persons who had lost the right arm could be trained to use the left. If children were thus trained, we would have a sturdier and healthier race, both mentally and physically.

